# When online learning becomes compulsory: Student nurses’ adoption of information communication technology in a private nursing education institution

**DOI:** 10.4102/curationis.v44i1.2152

**Published:** 2021-10-28

**Authors:** Petra Bester, Karlien Smit, Maryke de Beer, Pieter H. Myburgh

**Affiliations:** 1 Africa Unit for Transdisciplinary Health Research, Faculty of Health Sciences, North-West University, Potchefstroom, South Africa; 2 School of Nursing Science, Faculty of Health Sciences, North-West University, Potchefstroom, South Africa

**Keywords:** information communication technology, ICT, barriers and enablers, student nurses, online learning

## Abstract

**Background:**

Integrating the use of information communication technology (ICT) in nursing curricula when preparing student nurses for the digital health future such as the sudden online learning as a result of the coronavirus disease 2019 (COVID-19) pandemic is vital. However, when student nurses in a South African private nursing education institution, struggled to complete obligatory online learning courses, nurse educators had to search for solutions.

**Objectives:**

To explore the barriers and enablers for ICT adoption by a diverse group of student nurses in a private nursing education institution in the Free State Province.

**Method:**

Following a qualitative, explorative, interpretive-descriptive design, student nurses were invited to participate. Based on all-inclusive, purposive sampling with inclusion criteria enabled selecting, a total of 17 participants who took part in three focus groups and written narratives. Transcribed interviews underwent thematic analysis with co-coder consensus. The study adhered to strategies to enhance trustworthiness.

**Results:**

Students shared their views related to ICT and online learning within their theory and practice training. Student nurses held positive, negative and contrasting views of ICT adoption and online learning. Actions to master ICT adoption and online learning are highlighted. Information communication technology brings a challenging interdependence between nurses and technology.

**Conclusion:**

Integration of ICT into nursing programmes is important. The enablers and barriers to ICT are described. Expose students to different technologies, especially using smart phones to search for (academic/non-academic) information. The adoption of ICT should enhance the learning process and facilitate deep learning. Students preferred online learning for self-assessment and described how they tried to master ICT and online learning. Information communication technologies in the clinical setting highlight the challenged interdependence between nurses and technology. Context-specific recommendations are proposed.

## Introduction

For preparing the future nurses for the digital world of health, it is imperative to integrate information communication technology (ICT) into training student nurses. This fact is further validated by the disruption causes by the coronavirus disease 2019 (COVID-19) pandemic in all walks of life, especially students who were forced to adapt to online learning (Teräs et al. 2020:863). This unparalleled impetus to online learning (Teräs et al. 2020:863), necessitates revisiting students’ adoption of ICT. Online learning (also referred to as eLearning) is inseparable from ICT. As Coopasami, Knight and Pete (2018:305) highlighted that for the first-year South African student nurses, the success of online learning is closely linked to technology and equipment. Nursing in a digital age requires integration of information technology (IT) into the larger body of knowledge in nursing and in addition training the student nurses to adapt to an IT-rich work environment (Gonen, Sharon & Lev-Ari 2016:1). In fact, the adoption of digital solutions is necessary to contribute to the new digital-based models of care (Golinelli et al. 2020:2) entering the healthcare sector globally. Raman (2015:663) argued that the integration of ICT into nursing curricula is necessary to prepare nurses for evidence-based patient care. Information communication technology is therefore a tool to be used in the classroom and in the clinical setting. Oermann (2015:55) reiterated that student nurses should be exposed to technology in their clinical work environment to bridge the gap between theory and practice.

In this study ICT refers to the rapid convergence between IT (the hardware and software utilised to store, process and retrieve data) and communication technology (CT) (the electronic systems that enable communications between individuals and groups) (Huang et al. 2012:35). Furthermore, it is also a three-layered convergence between the cloud (the Internet and broadcasting services); the pipe (traditional telecommunications networks and cable networks); and the devices (personal electronic devices) (Huang et al. 2012:36). Information communication technology provides considerable benefits in achieving health goals; demonstrating what health outcomes are reached at what cost. In healthcare, ICT is utilised in electronic health records (eHealth records); routine health (e.g. web-based surveillance systems); vital registrations (e.g. births and mortality); consumer health informatics (e.g. self-management systems); mHealth apps (e.g. smart phones); telemedicine (e.g. ICTs for healthcare or – education distantly); virtual healthcare (e.g. a doctor consults the patient via Zoom) and health research (Hanna 2016:119). The National Digital Health Strategy of South Africa 2019–2024 referred to the above as digital health and digital health technologies (National Department of Health 2019:11). The Technology Acceptance Model (TAM) was developed in 1989 by Davis (from information systems), and presents a useful theoretical approach to understand why workers in general are unwilling to use ICT, despite having the available technology (Davis, Bagozzi & Warshaw 1989:982). The TAM outlines people’s behavioural intention to use technology being influenced by their attitude and general impression thereof. Davis et al. (1989) proposed that all users exposed to a new technology should be presented with a perceived usefulness (PU) and perceived ease-of-use (PEOU). In the context of nurses in a hospital unit, PU implies whether nurses perceive that the digital thermometer will measure patient’s temperature accurately; and PEOU means if this digital thermometer will be easy to use by all the nursing staff. User-friendly technology enables the use thereof.

The integration of ICT into higher education is a global phenomenon. Worldwide ICT is viewed as the vehicle to facilitate educational reform and develop communication platforms (Adarkwah 2020:1). Within the modern society, ICT has become the basic building block for education as it is the essential skill required for reading, writing and numerals (Penaflor-Espinosa 2017:167). Even more so in nursing, when training nurses to have critical thinking skills and forward-thinking knowledge to make precise choices in life-threatening situations (Penaflor-Espinosa 2017:167). Both the Global South and North invest in health IT (Lulin et al. 2020:1). In South Africa, the Strategic Plan for Nurse Education, Training and Practice 2012/2013–2016/2017 called for the improved use of ICT in nursing and midwifery care (National Department of Health 2013:11). It is essential to enhance student nurses’ access to ICT in education and training to support their learning whilst increasing access to enhanced nursing practice needs; and it is the employers responsibility to provide these technologies (National Department of Health 2013:48, 74). ICT competencies are to be incorporated into nursing curricula (National Department of Health 2013:50). In Ghana, all health education programmes must provide a compulsory basic practical ICT skills training to support eHealth (Frimpong, Asare & Otoo-Arthur 2017:34). In Nigeria (Irinoye, Ayamolowo & Tijnai 2016:8), although ICT is viewed as an essential tool to improve education quality, the majority of nurses do not have formal computer training and do not even own a personal computer. In South-East Asia (Nwozichi et al. 2019:1), insufficient attention is granted to the integration of ICT into specifically nursing education. Penaflor-Espinosa (2017:176) stated that ICT must integrate into the comprehensive student nurse learning environment in the Philippines. In Saudi-Arabian universities, a positive correlation exists between ICT adoption and academic performance (Basri, Alandejani & Almadani 2018:1) although students lacked laptops and smart phones because of insufficient Internet access and high costs (Basri et al. 2018:7). Mather, Cummings and Gale (2019:1) confirmed that in Australia, nurses are insufficiently prepared for the digital future – a digital reality that is already arriving in the healthcare environment. Part of this unpreparedness include those nurses who do not recognise their leadership role in the decisions towards digital technology adoption. In Minnesota, United States of America, the adoption of health IT is essential to enhance quality patient care (Murray 2015:1).

Within South Africa various factors are listed for poor adoption of ICT by student nurses. Harerimana and Mtshali (2018:1) reported nurses’ underutilisation of the internet for academic and non-academic use. These nurses reported restricted Internet access; extremely slow internet connections; not enough computers and inadequate training on how to utilise Internet facilities, because of different reasons. Coopasami et al. (2018:305) confirmed that although student nurses value online learning, they lack computer skills and hardware and should be supported with these insufficiencies if they are to enhance their attitudes towards online learning. In KwaZulu-Natal, mobile devices are a functional professional and educational tool for student advance midwives (Chipps et al. 2015:1). These nurses regarded their own technology competence as low and required support from their nursing education institution to develop their mobile network literacy skills and optimise their use of mobile phones.

### Research question

Expounded from the literature above, this article set out to answer ‘What are the barriers and enablers for ICT adoption amongst student nurses in a private nursing education institution in the Free State, South Africa?’.

### Research aim and objectives

To explore the barriers and enablers for ICT adoption by a diverse group of student nurses in a private nursing education institution in the Free State Province.

### Problem statement

The private institution provided compulsory online learning courses that had to be completed in the classroom. Lecturers initially welcomed this technology because of the value proposition to strengthen the ICT integration. Student nurses however, presented with a negative attitude and a lowered pass rate. They highlighted the barriers and enablers for ICT adoption and online learning within the private nursing education institution. They shared how they used online learning and mastered ICT adoption and amplified the importance of exposure to technology. The student nurses preferred online learning for self-assessment and warned against superficial learning. Information communication technology in the clinical setting implies an interdependence between the nurse and technology in the digital world of nursing.

## Research methods and design

A qualitative, explorative, interpretive-descriptive design was followed to identify the barriers to and enablers of ICT adoption by student nurses in a private nursing education institution in the Free State, South Africa. The nursing programmes include 1 year of training for an auxiliary nurse, referred to as a pupil nurse; and 2 years of training leading to registration as an enrolled nurse. Enrolled nurses apply for a 2-year bridging course, leading to registration as a general nurse. Inclusion criteria comprised of (1) a registered pupil-enrolled nurse or bridging nurse student at the private nursing education institution between 01 January 2015 and 31 December 2015; (2) willing to provide written, informed consent to participate voluntarily in focus groups (FGs) to be digitally voice-recorded and to be used for writing narratives; and (3) communicate in English as mode of instruction. Through purposive sampling (Botma et al. 2010:124), 17 student nurses aged between 18 to 46 years, from different indigenous cultures and language groups, participated in the research.

Data collection was realised in two phases. Phase 1 explored barriers to and enablers of ICT adoption, through three digitally voice-recorded FGs. The compilation of the three FGs were as follows: six participants in FG 1, five participants in FG 2 and six participants in FG 3. Within and across, group saturation was reached. Focus groups were conducted in the seminar room at the private nursing education institution. Each FG lasted approximately 1 h.

The question asked in the FG included: (1) What do you see as ICT? (2) How do you view the use of ICT in your studies? (3) What prevents (barriers) you from using ICT in your studies? and (4) What can help (enablers) you increase the use of ICT in your studies? Phase 2 explored participants’ individual experiences through narratives, giving more insight into their diverse perspectives. This article presents the results from the FGs. Field notes (methodological, theoretical and personal) (Polit & Beck 2008:406–407) provided a written description of what was seen, felt, heard, thought and experienced during the research process.

Goodwill permission was attained from Company A – one of the largest private hospital groups in South Africa (pseudonym to protect the company’s identity). A mediator, an administrative assistant at the private nursing education institution, recruited participants and an advertisement was posted on the students’ notice board, inviting them to participate. The mediator explained informed consent at least 24 h before data gathering. To prevent bias and coercion, the researcher (a nurse educator at the identified nursing education institution) had no direct contact with the participants. The researcher maintained participants’ anonymity and confidentiality externally by using codes, safekeeping the data in hard copy and digital format. An independent interviewer with a master’s degree in Health Science Education conducted three FGs of approximately 1 h each.

All 17 participants wrote a narrative after the FGs. Digitally recorded voice notes from the FGs were transcribed and checked for accuracy. The transcripts and narratives thematically analysed following Creswell’s six-step method of data analysis that included: (1) organising and preparation of data; (2) developed a sense of all the data; (3) coded data following the nine steps of Tesch; (4) identified and described themes; (5) representation of the findings; and (6) interpretation of the data (Creswell 2009:184). Trustworthiness (Lincoln & Guba 1985:218) was enhanced by pursuing truth value through credibility with prolonged engagement and reflection; applicability through transferability by providing a thick description of the full research process; consistency through dependability as evidenced by a detailed audit trail; and neutrality through confirmability by means of reflexivity.

## Results

### Demographic profile

The demographic profile of the participants revealed that the average age of participants was 27.8 years, whilst the average age of newly registered nurses (all categories) according to the South African Nursing Council (SANC) is 33 years (SANC 2015). Besides one male student, the rest of the participants were all female. The information provides context and supports the demographic profile of the current study. Nursing as a profession in South Africa tends to be female-dominated with less males. This study were also female-dominated with less male participants. The participants spoke five of the 11 official languages in South Africa; the majority spoke Afrikaans. Of the total participants, 70.5% were in their second year of study. Refer to Table 1 for the demographic information of the participants.

**TABLE 1:** Demographic data of participants (*N* = 17).

Criteria	Group 1 (*n* = 6)	Group 2 (*n* = 5)	Group 3 (*n* = 6)
**Age**			
(Mean age in years)	24.3	31.6	27.6
20–25 years	4	1	2
26–30 years	2	1	3
31–35 years	-	2	-
36–40 years	-	-	1
45–50 years	-	1	-
**Gender**			
Male	1	-	-
Female	5	5	6
**Culture**			
Black	3	2	2
White	2	3	3
Coloured	1	-	1
**Language**			
English	1	3	-
Afrikaans	2	2	4
Sotho/Southern Sotho	2	-	1
Zulu	1	-	-
Xhosa	-	-	1
**Programme enrolled**			
Pupil enrolled nurse 1st year	1	1	2
Pupil enrolled nurse 2nd year	2	2	2
Bridging course 1st year	1	1	-
Bridging course 2nd year	2	2	2

### Themes, categories and sub-categories

**FIGURE 1:**
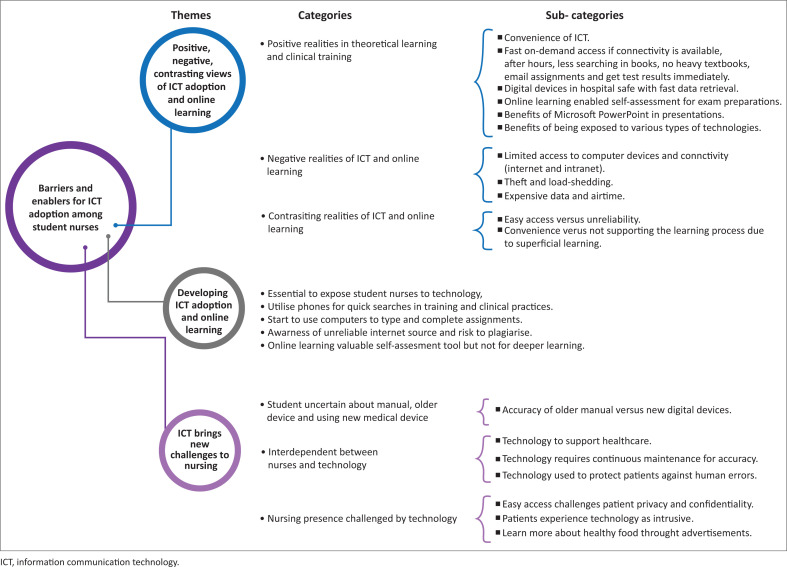
Themes, categories and sub-categories of the barriers to and enablers for ICT adoption by student nurses.

Figure 1 presents the 3 themes, 11 categories and 18 sub-categories that emanated from the data.. The themes are discussed in the following paragraphs and the participant number and FG number are indicated with direct quotes. In exploring the barriers to and enablers for ICT adoption, student nurses presented with positive, negative and contrasting realities of ICT and online learning. However, the student nurses also proposed how to not only adopt to ICT and online learning but master it. The student nurses didn’t differentiate between teaching-learning and clinical care technologies but viewed it as an integrated whole and highlighted also how ICT brings new challenges to consider in nursing.

#### Theme 1: Positive, negative and contrasting realities of ICT and online learning

Participants shared several realities of ICT and online learning and didn’t confine it only to their theoretical learning but also to their training in the hospital where student nurses do their clinical practice work.

The *positive realities* of ICT related to convenience and fast on-demand access to information, subjective to connectivity. The participants explained that convenience implied having lightweight ICT devices such as smart phones, which facilitated access to information anywhere and anytime. Convenience also meant access to information from home after working a 12-h shift. The participants explained that they don’t have to travel to libraries or spend hours searching for specific information anymore, as one participant explained:

‘… [*B*]ecause sometimes you are … working from seven to seven so we don’t have time to go to the libraries, to go to book shops to find information. So, it saves time …’ (Participant 5, FG 2)

Furthermore, the participants elucidated that information is immediately and on-demand accessible from their phones. As one participant explained that her phone is the first source to consult when she is confronted with an unknown diagnosis:

‘I go to my phone and Google it [*unknown diagnosis*].’ (Participant 1, FG 1)

The participants found browsing the internet user-friendly, especially when using the web services of Google, compared to referring to textbooks and visiting libraries. As participants from the second FG reported:

‘You don’t have to go through all the books, all the library books to get just this little bit of information.’ (Participant 1, FG 2)

‘You get information easily because if you want to find something you just Google it and ja (yes) so easy to get information.’ (Participant 5, FGD 2)

Another participant said they could email assignments and complete online assessment activities on the intranet, the localised and restricted private network available for students and staff within the private hospital group where they are trained and/or employed. Receiving immediate results from online assessments gave students on-the-spot feedback on their own learning:

‘Once you’ve complete the questionnaire, you get the feedback immediately. They are computerised, you don’t have to wait a day or two on that project that you did on the computer.’ (Participant 2, FG 1)

‘… get your results quicker.’ (Participant 2, FG 3)

Participants explained that online learning enabled their preparation for exams because they could evaluate their knowledge and check their calculations in pharmacology. Online quizzes assisted participants to determine how much revision and extra study they needed:

‘… [*W*]ith the E–learning, they explain to you what you must do, how you must do it and what more or less will be in the test.’ (Participant 1, FG 2)

‘… [*I*]t (online assessments) has questions that are constructed in a way that we are going to get them in an examination. Like for instance, when we did our medication we did the E-learning, we had calculations and all that. So that truly helped us a lot because the things we thought we knew we actually didn’t. The way, I remember it was a calculation [in pharmacology] we thought we actually did right but then at some point we did wrong because of the units that we were using.’ (Participant 5, FG 1)

Participants associated efficiency of digital devices not only with online learning but also with in-hospital care. The participants described digital devices as safe to use; the memory function could store patient clinical information which could be retrieved if they missed writing it down immediately. As one participant explained:

‘… [*B*]ecause the (patients’) vital information are being stored it’s easier to just go back.’ (Participant 4, FG 1)

Another positive reality of ICT and online learning was the use of various applications, such as Microsoft PowerPoint slides, which improved clinical facilitation and lecturing in a large class settings especially when students have limited time to make class notes. In the past the student nurses had to write posters by hand, but by using PowerPoint, they could share patient case studies faster with more students. One participant explained that one can:

‘Present it (patient case studies) for everybody and that whatever you are presenting orally, you can also demonstrate on the PowerPoint.’ (Participant 5, FG 1)

Besides the positive realities of ICT and online learning, the participants also highlighted the *negative realities*. The first negative reality is that although all the student nurses had mobile or smart phones, they neither possessed nor had access to tablets or laptops to prepare assignments. Phones can be used for internet searches and communication, but the students required a tablet, laptops or computers to be able to type and complete the assignments:

‘Not all of us have laptops, not all of us have internet. We’ve all got cell phones, but they can’t do a whole project on a cell phone. So, to type the project and to go onto internet to find the information becomes a struggle if you don’t have the laptop and the internet, and so forth.’ (Participant 3, FG 1)

Because the online learning programmes and the limited computers that were available to access these programmes were accessible only through the Company A’s intranet, students could not access the online learning platform outside their workplace or from home. As participant explained:

‘On the other hand, it is difficult because like participant number 3 said you don’t have the access to the computers and especially in the work situation, there is only a few computers that you are allowed to use and it’s not always available. So, it makes it difficult because it is not that you can go and do your E-learning because it’s mostly available on the internet. So, it’s difficult to almost get access to that, to do the E-learning.’ (Participant 4, FG2)

Another participant confirmed that:

‘Access to the internet is very difficult.’ (Participant 2, FG 3)

Connectivity albeit from the internet, Wi-Fi, intranet or accessing networks via one’s phone, remains a great challenge to the participants. There are various reasons for this limited connectivity. The majority of participants don’t have internet access at their place of residence and cannot continue to work online afterhours at home:

‘I think the availability of the internet is a problem because not all of us have it available to us …’ (Participant 3, FG 1)

Other reasons for limited connectivity were theft and intermittent electricity load-shedding schedules, prominent in South Africa, which also impede access to ICT. Data and airtime are expensive and unaffordable for many student nurses:

‘The negatives might be yes we’ve got load shedding, we’ve got theft.’ (Participant 3, FGD1)

‘[*T*]he internet when using it on your tablet or mobile, it’s very expensive. You don’t have your uncapped internet. So, I think that could also become a barrier point at some stage because some people just won’t be able to afford it.’ (Participant 1, FGD1)

The participants shared realities that had both positive and negative aspects, referred to as the *contrasting realities* related to ICT and online learning. Participants described that although information is easily accessible through the web, the scientific unreliability thereof, especially from non-academic websites, is concerning:

‘We find sources and some of the sources are not reliable. For instance, with like Wikipedia …’ (Participant 5, FG 1)

‘… [*W*]e aren’t allowed to use it [*Wikipedia*] because anyone can add information on a certain website …’ (Participant 1, GD 2)

Furthermore, participants explained that whilst ICT and online learning are convenient, one might become too lazy to research and read books. Also, online learning might lead to superficial learning:

‘I think it makes us lazy because the internet is here you get everything there and the information is there. Our assignments we don’t go to the library to find books to go through every page. So, it makes us lazy.’ (Participant 5, FG 2)

Participants explained that they used the internet to obtain quick answers for immediate questions. However, the participants acknowledged that they didn’t fully grasp what they were supposed to learn in the same manner that they would have if they had to assimilate information from a hardcopy book. Participant acknowledged that everything is faster electronically, but also stated that:

‘… [*I*]t’s difficult to grasp concepts.’ (Participant 2, FG 3)

‘When on the internet, … I think for me personally you get the feeling that you understand and that you adapt quickly more quickly compared to the information that you get on the internet because you just browse through everything where with the books there is more focus in terms acquiring information. With the internet you just tap a word, and you get all these other information …’ (Participant 2, FG 1)

‘… there is too much information that is accessed at a particular point compared to when you have a book.’ (Participant 2, FG 1)

With the benefits of speed, on-demand access and ease-of-use of the internet for online learning, participants highlighted the negative side also. This is the side of being exposed to too many information and superficial learning:

‘… [*T*]hen the student would just go there and just write anything that is there without understanding exactly what’s going on. So, your assignment or project might be right and get all the full marks and stuff, but you personally won’t understand what the content is about. That’s my problem also about it.’ (Participant 6, FG 1)

#### Theme 2: Developing information communication technology adoption and online learning

The second theme described how participants developed their ICT and online learning skills. Participants explained the importance of being exposed to technology. Techno-exposure is introducing student nurses to technology in their teaching and learning and in the clinical practice. By exposing student nurses to technologies, it enables them to experience the use of these technologies regularly in healthcare and training:

‘… [*S*]he is an adult learner and that she wasn’t exposed to all this technology but now that she is doing E-learning, that gave her the courage, the oomph to go on because she must do her things a certain way and because she wants her qualification.’ (Participant 3, FG 2)

The participants described how they utilise their phones as the go-to device to do quick searchers. The participants explained that they used their phones in their training and in the context of the healthcare to browse the internet. The participants explained that they used their phones in their training and in the context of the healthcare to browse the internet to find quick references:

‘I think we could get quick references … I go to my phone and Google it. So, we don’t have to wait …’ (Participant 1, FG 1)

‘Cell phones are used to access information.’ (Participant 5, FG 3)

Participants explained that they started to use computers and laptops to type and complete assignments. In general, it seemed as if the participants all were active users of their phones but didn’t have the basic computer and typing skills when they enrolled for their nursing training:

‘I never had background in school like of using a laptop or whatever. I was only exposed to that when I started learning.’ (Participant 3, FG 2)

The participants seemed to be aware of the vast array of information provided on the internet and that not all information is valid and trustworthy. They also noted the propensity to plagiarise when using internet information sources:

‘We find sources and some of the sources are not reliable.’ and ‘… we aren’t allowed to use specific websites soos by voorbeeld [*for example*] Wikipedia. We aren’t allowed to use it because anyone can add information on a certain website.’ (Participant 5, FG 1)

‘Also with the internet usage, plagiarism is more where some of the educators wouldn’t know the site.’ (Participant 6, FG 1)

Finally, participants voiced that online learning was a valued self-assessment tool for exam readiness, but the participants had contradictory views of ICT and online learning as learning tools. The structured nature of the online learning and the timeous feedback from the online learning were appreciated. It guided students to know where they are in the lesson and gave them feedback on their current knowledge levels. However, some participants did voice that a deeper level of learning happened when they had to acquire information using books:

‘To me it makes it easier because with the E–learning they give you, they explain to you what you must do, how you must do it and what more or less will be in the test. So if you have any questions or anything you can run through your books because you will know where in the books it is. It’s multiple questions which are also easier to just comprehend and it’s faster.’ (Participant 1, FG 2)

‘E-learning teaches you by pictures and diagrams.’ (Participant 5, FG 3)

‘When on the internet, the weight of it becomes lesser but when you physically go look for it in the books I think for me personally you get the feeling that you understand and that you adapt quickly more quickly compared to the information that you get on the internet because you just browse through everything where with the books there is more focus in terms acquiring information.’ (Participant 2, FG 1)

#### Theme 3: Information communication technology brings new challenges to nursing

Participants seemed uncertain about when to use technology or continue with manual nursing activities. The participants explained that technology brings ambiguity regarding accuracy. Older medical devices are more trusted by older medical practitioners, whilst digital devices are fast and more convenient. However, digital medical devices might also be inaccurate if not properly charged. Participants stated:

‘Always keep up to date on the manual ways of doing stuff.’ (Participant 5, FG 3)

‘Some Doctors still prefer the BP (blood pressure) taken manually. They feel it’s more accurate and I encounter this problem with the electronic system where you find on the manual reading, where the glucose is four pluses where on the machine itself, is two pluses for the glucose in the human. There is always a variance between the manual and electronic gadgets … For us as nurses specifically might lead us to address a patient wrongly or treat the patient wrongly.’ (Participant 2, FG 1)

Participants explained the interdependence between nurses and technology. Technology should be an instrument that supports healthcare of patients and requires continuous maintenance to ensure its accuracy. They explained that although technology can speed-up activities and improve convenience for nurses, its reliability remains dependent on nurses’ maintenance:

‘So, if the people don’t take care of the machine’s, from day one, it can in the end give you some problems.’ (Participant 1, FG 1)

Furthermore, participants described that technology is also applied to protect patients against the human error. The participants continued that technology can be used with a digital audit trail of backed-up data per patient, when there is mistrust about the competence and integrity of nurses:

‘They (nurses) get to a patient and they in a hurry, so they don’t really count the respiration. Hulle skat (they estimate), So, it’s going to be more accurate if we are able to do things more electronically if we could get something to electronically read the respiration and the temperature that is accurate.’ (Participant 3, FG 1)

‘Instead of having an abnormal patients BPM out of range, we will just take and write down whatever they feel is normal … I think if it’s uploaded on the patients profile anyone could see the reflection from the readings and those that have been noted down.’ (Participant 2, FG 1)

It was interesting when participants explained that a caring nursing presence can be challenged within a technological environment:

‘I think the one thing that we must be careful of is that we don’t totally move away from the patient because after all nursing is that touch and caring and that. So, I think that is one thing that we must be very careful about.’ (Participant 1, FG 1)

Patient privacy and confidentiality are challenged when technology makes patient information easily available to all nurses:

‘As long as I know your surname and your name I can just go into the system and get your results.’ (Participant 3, FG 2)

This applies to both health information systems and social media that can:

‘… [*I*]nvade(d) people’s privacy.’ (Participant 3, FG 2)

Participants agreed that patients may experience technology as intrusive to their care:

‘… [*T*]he patient is a first time in the hospital doesn’t know our gadgets; they will have a fright of a life time … So, you will have an increase of blood pressure and everything and it will affect the patient negatively because they will actually feel bad, and they will feel unsafe in the environment.’ (Participant 1, FG 2)

## Discussion

Student nurses perceived ICT as a tool for quick and easy access to information (Sá, Nabais & Oliveira 2019). According to Statistics South Africa as of January 2021, 38.13 million South Africans (of the approximate 59 million population) were active internet users of which 36.13 million were active mobile internet users (Statistics South Africa 2021). Despite Internet usage growth, South African mobile Internet users are dissatisfied with slow broadband Internet speeds and unreliable service (Staff Writer 2021). Mobile devices are a preferred tool for advanced midwives in both their teaching and professional responsibilities in KwaZulu-Natal (Chipps et al. 2015:1), despite contextual challenges. Poor access to networks is a barrier to ICT adoption by nurses in South Africa (Nkosi, Asah & Pillay 2011:876), although South African nurses are keen to become accomplished ICT users (Mapi, Dalvit & Terzoli 2008:85).

It is however imperative that nursing education institutions support students in providing ICT infrastructure (hardware) and develop student nurses’ computer skills (Coopasami et al. 2018:305). Higher education institutions are adopting ICT to enhance education as directed by the National Plan for Higher Education and the Draft Paper on e-Education (2003) (as in Ravjee 2007:29). Student nurses did view that ICT and online learning facilitated the learning process and contribute to their decision-making abilities (Sá et al. 2019) both in the classroom and the clinical work environment. Surprisingly, the internet-generation nurses don’t hold an automatically positive view of technology-based healthcare (Van Houwelingen et al. 2017:717).

Information communication technology should enhance the learning process of student nurses. Superficial learning involves cognitively passive learning, in contrast to cognitively active deep learning (Weimer 2012). Deep learning and organising comprehensive events into one long-term memory can be hindered by cognitive loading, such as an overload of over-stimulating web-based information (Stanger-Hall 2012:1). Online learning can be applied as a useful self-assessment tool when knowledge evaluation is the end goal (Mettiäinen 2015:42) and can be useful in teaching nurses to conduct self-assessments (Smith et al. 2010).

Health information systems with insufficient data-capture rules, alarm fatigue, combined with human error, multitasking and high workloads, could become unreliable (Wachter 2015). Digital medical equipment should be managed with caution, given its sensitivity. For example, the reliability and accuracy of a Dinamap 8100 (digital blood pressure machine) evaluated against international criteria concluded that systolic blood pressure measurements were accurate, but diastolic pressures were not (Heinemann et al. 2008:1). Furthermore, patient data security should be guaranteed because unauthorised access might be possible (Singh & Muthuswamy 2013:1533). Techno-ethics in nursing requires further exploration (Korhonen, Nordman & Eriksson 2015:561) because nurses are the interpreters of technology for their patients (Korhonen et al. 2015:572).

Three FG discussions with a diverse group of student nurses enabled within and across group saturation. Although the interview schedule questions focused on barriers and enablers, student nurses’ response to ICT adoption wasn’t limited to the classroom only but included all technology in the clinical setting as well. Also, it was challenging to accommodate student nurses who had to do clinical work as well, into the FGs that suited them and the interviewer. For future reference, synchronised FG discussions might bring in a larger number of participants. However, the mediator supported the researcher to schedule appointments with students at times when the majority of them were available.

It is recommended to integrate ICT meaningfully into nursing curricula to facilitate deep learning from an approach of learning through instruction and not learning with instruction. With improving access to uncapped Internet in South Africa, employers could provide monitored access to the Internet, be it at a designated communal area (i.e. staff rooms) or Wi-Fi Internet access for use on personal mobile phones. In doing so, it would remove some of the barriers experienced by this cohort, and likely most South African nurses, and enable active learning by means of continuous access to information as questions may arise. Start with using online learning as a self-assessment tool for student nurses who can develop towards a deeper learning process. With the rapidly changing technological landscape towards new technologies (such as extended realities and artificial intelligence), it would be best to bridge technological gaps earlier rather than later.

## Conclusion

This study confirmed that ICT must be integrated into the teaching, learning and clinical work environment of student nurses because ICT is closely linked to online learning. ICT adoption enablers are: convenience; fast and on-demand access to information; memory function of devices; self-assessment and being exposed to technology. Barriers to ICT adoption included: insufficient access to devices and connectivity; contextual challenges such as costs, load-shedding and crime. Contradictory views about ICT adoption were that ICT was convenient but supported superficial learning and that ICT was user-friendly but not always accurate. Ensure that ICT is utilised meaningfully to enhance the learning process (learning through instruction) and facilitate programme outcomes. Student nurses require affordable, stable connectivity outside the training institution’s/workplace’s infrastructure. ICT skills and online learning develop through technology exposure, using phones to browse the Internet and computers to do assignments. Student nurses preferred online learning for self-assessment and exam preparation. Within their clinical setting, student nurses were uncertain about the accuracy of older, manual versus digital technologies, and concerned about caring presence of nurses in technology-driven healthcare environments.
